# Evidence for a topological excitonic insulator in InAs/GaSb bilayers

**DOI:** 10.1038/s41467-017-01988-1

**Published:** 2017-12-07

**Authors:** Lingjie Du, Xinwei Li, Wenkai Lou, Gerard Sullivan, Kai Chang, Junichiro Kono, Rui-Rui Du

**Affiliations:** 1 Department of Physics and Astronomy, Rice University, Houston, TX 77005 USA; 2 Department of Electrical and Computer Engineering, Rice University, Houston, TX 77005 USA; 3 SKLSM, Institute of Semiconductors, Chinese Academy of Sciences, Beijing, 100083 China; 4 Teledyne Scientific and Imaging, Thousand Oaks, CA 91630 USA; 5 Department of Materials Science and NanoEngineerng, Rice University, Houston, TX 77005 USA; 6 ICQM, Peking University, Beijing, 10083 China

## Abstract

Electron–hole pairing can occur in a dilute semimetal, transforming the system into an excitonic insulator state in which a gap spontaneously appears at the Fermi surface, analogous to a Bardeen–Cooper–Schrieffer (BCS) superconductor. Here, we report optical spectroscopic and electronic transport evidence for the formation of an excitonic insulator gap in an inverted InAs/GaSb quantum-well system at low temperatures and low electron–hole densities. Terahertz transmission spectra exhibit two absorption lines that are quantitatively consistent with predictions from the pair-breaking excitation dispersion calculated based on the BCS gap equation. Low-temperature electronic transport measurements reveal a gap of ~2 meV (or ~25 K) with a critical temperature of ~10 K in the bulk, together with quantized edge conductance, suggesting the occurrence of a topological excitonic insulator phase.

## Abstract (web summary)

Weakly bound electron–hole pairs may condensate in two-dimensional systems, but experimental evidence has been lacking. Here, Du et al. report optical spectroscopic and electronic transport evidences for the formation of an excitonic insulator gap in topological InAs/GaSb quantum wells.

## Introduction

**Fig. 1:**
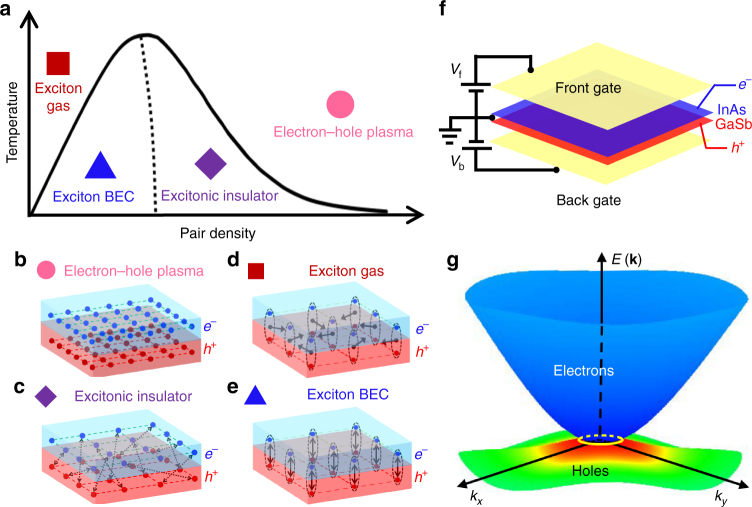
Excitonic insulator in inverted InAs/GaSb quantum wells tuned by double gates. **a** Phase diagram for an electron–hole (e–h) system in the parameter space of temperature and e–h pair density. **b** Pink circle: an e–h plasma (or a semimetal). **c** Purple diamond: the excitonic insulator, where electrons and holes are weakly bound, like Cooper pairs. **d** Red square: an exciton gas consisting of bosonic particles with a finite center-of-mass momentum. **e** Blue triangle: exciton Bose–Einstein condensate, where the exciton states are degenerate. In **b**–**e**, the blue dots represent electrons, the red points are holes, the dashed ellipses indicate the strong binding between electrons and holes, the dashed lines with arrows mean the weak binding between electrons and holes, and the single arrows show the center-of-mass movement of excitons. **f** Sketch of a device with front and back gates. The back-gate is used to fix *p*_0_, and sweeping the front-gate voltage allows us to find a resistance peak at the charge-neutral-point, *n*_0_ ~ *p*_0_. **g** Band structure of InAs/GaSb quantum wells calculated using the 8-band **k**·**p** method, for low *n*_0_

It was predicted several decades ago^1,2^ that Coulomb interactions in an electron–hole (e–h) co-existing system can make the normal semimetallic state unstable against the spontaneous appearance of excitons, or bound e–h pairs, inducing a phase transition into an insulator, called the excitonic insulator (EI) or Bardeen–Cooper–Schrieffer (BCS)-like excitonic condensation. The EI phase emerges below a density-dependent critical temperature (Fig. 1a), where a gap opens at the energy of the original Fermi surface of the semimetal, in a manner analogous to the BCS gap in a superconductor^3–9^. In the density regime where the EI phase is expected to occur from semimetal (Fig. 1b), the spatial extent of the exciton wavefunction is larger than the average inter-exciton distance, i.e., electrons and holes are only weakly bound (Fig. 1c), similar to Cooper pairs. Conceptually, this density regime is distinct from the dilute limit where a quantum-degenerate gas of tightly bound e–h pairs (Fig. 1d) is transformed into a Bose–Einstein condensate (BEC) with macroscopic coherence (Fig. 1e). The possibility of excitonic ground state formation is enhanced in two-dimensional (2D) systems due to reduced screening. There have been extensive experiments on the possible appearance of nonequilibrium BEC in photoexcited 2D e–h systems probed by photoluminescence^10,11^, and equilibrium BEC state in quantum Hall electron-electron double-layers probed by counter-flow measurements^12^ and light scattering^13^. Note that these two types of BEC states have distinctions, besides the way to prepare BEC state, the former state is a short lived metastable superfluid supporting interference and vortices, whereas the latter is a ground state of the system. However, the EI side of the phase diagram has not been experimentally explored in any 2D system.

The originally proposed EI was based on a low density, equilibrium e–h gas that exists in certain semimetals, and the possibility of the EI phase was systematically studied in the vicinity of a pressure-tuned semimetal-semiconductor transition^5^. Highly controllable 2D semiconductor materials emerged in ensuing years. In particular, InAs/GaSb quantum wells (QWs) exhibit unique inverted band structure with finite overlap of the conduction and valance bands, allowing the coexistence of spatially separated electrons and holes without photoexcitation, which offers a natural setting for equilibrium excitons and consequent formation of condensates including the EI phase^9,14,15^. Electrons are located in the InAs QW and holes are located in the GaSb QW, and thus, they are spatially separated in real space. The average separation can be defined as one half of the thickness of the double-QW structure. In this system, signatures of magneto-excitons were previously reported^16^. Recently, the quantum spin Hall (QSH) effect was explored in such QWs^17–20^. Robust quantized edge transport was observed^19^, persisting even at strong magnetic fields and high temperatures; it was theoretically suggested^20^ that the formation of a topological EI^21^ state may account for these unexpected properties. A topological EI in 2D has helical edge states propagating on the perimeters of a bulk EI state.

Here, we report optical spectroscopic and electronic transport evidence for the appearance of a BCS-like excitonic insulator gap in gated InAs/GaSb QW devices (Fig. 1f) at low temperatures with a low intrinsic e–h pair density, *n*_0_ ~ *p*_0_ ~ 5.5 × 10^10^ cm^−2^ (we use *n* and *p* to denote, respectively, the band electron and hole densities, and in particular, *n*_0_ and *p*_0_ denote the electron and hole densities at the charge-neutrality point). Our temperature- and magnetic field-dependent terahertz (THz) transmission spectroscopy data can be quantitatively explained through our calculated pair-breaking excitation dispersion, *E*(k), of the presumed EI state formed in the system. Complementarily, our low-temperature electronic transport measurements also suggested the existence of a gap, determining the gap energy to be ~2 meV (or ~25 K). We found the gap value to be roughly independent of the strength of an applied in-plane magnetic field but close quickly with increasing *n*_0_ or temperature. Together with theoretical analysis, we interpret these results as the observation of EI gap opening in this 2D equilibrium e–h gas, suggesting that a 2D EI phase is realized in our QWs. Moreover, the system exhibited helical edge transport behavior, which supports the notion that the observed low-temperature e–h phase is a topological EI.

## Results

### Devices

Our devices were made from inverted InAs/GaSb QWs grown by molecular beam epitaxy^18,19^ (also see Methods and Supplementary Figs. 1 and 2). Transport measurements were performed on wafer A and C, in which a conducting GaAs or GaSb substrate serves as a back-gate. In this structure (Fig. 1f), the back-gate bias voltage (*V*_b_) was limited to the negative range, *V*_b_ ≤ 0; starting with *V*_b_ = 0 and with increasing negative bias, we were able to introduce more holes (*p*) in GaSb QW. Correspondingly, we could sweep the front-gate bias *V*_f_ (for electrons in InAs QW) and reach the charge-neutrality point (CNP), i.e., *n*_0_ = *p*_0_. The lowest *n*_0_ in our devices was ~5.5 × 10^10^ cm^−2^, which was achieved at *V*_b_ = 0. Figure 1g shows the band structure of InAs/GaSb QWs calculated using the 8-band **k**⋅**p** method for low *n*_0_ at *V*_b_ = 0. The average inter-exciton in-plane distance, 2*r*_avg_, corresponding to *n*_0_ ~ 5.5 × 10^10^ cm^−2^, defined through 1 = *n*_0_·*πr*_avg_^2^, is ~48 nm. This value should be compared with the effective Bohr radius, *a*_B,_ which is estimated to be ~30 nm within a simple effective-mass approximation^15^ using the following parameters^22^: electron effective mass $m_{\mathrm{e}}^*$ ~ 0.032*m*_0_ (*m*_0_ = 9.11 × 10^−31^ kg), hole effective mass $m_{\mathrm{h}}^*$ ~ 0.136 *m*_0_, dielectric constant *ε* ~ 15, and interlayer distance between the centers of the electron and hole wells *d* ~ 10 nm. Therefore, we have *r*_avg_/*a*_B_ ~ 0.8, indicating strong wavefunction overlap between excitons, a situation reminiscent of Cooper pairs.

### Theoretical model

The EI state is expected to have distinctly different optical and transport properties from an exciton BEC. Our system is described by the Hamiltonian^3,7–9^:

1 $$	{\hat {\it{h}}_{{\mathrm{e - h}}} = \mathop {\sum}\limits_{\mathrm{k}} {\left( {{\it{E}}_{\mathrm{k}}^{\mathrm{e}}{\mathrm{a}}_{\mathrm{k}}^{\mathrm{\dagger }}{\mathrm{a}}_{\mathrm{k}}{\mathrm{ + }}{\it{E}}_{\mathrm{k}}^{\mathrm{h}}{\it{b}}_{\mathrm{k}}^{\mathrm{\dagger }}{\it{b}}_{\mathrm{k}}} \right)} }\\ 	{{{\mathrm{ + }}\frac{{\mathrm{1}}}{{\mathrm{2}}}} \mathop {\sum}\limits_{{\mathrm{k,k{\prime},q}}} {\left( {{\it{V}}_{\mathrm{q}}^{{\mathrm{ee}}}{\mathrm{a}}_{{\mathrm{k + q}}}^{\mathrm{\dagger }}{\mathrm{a}}_{{\mathrm{k{\prime} - q}}}^{\mathrm{\dagger }}{\mathrm{a}}_{{\mathrm{k{\prime}}}}{\mathrm{a}}_{\mathrm{k}}{\mathrm{ + }}{\it{V}}_{\mathrm{q}}^{{\mathrm{hh}}}{\it{b}}_{{\mathrm{k + q}}}^{\mathrm{\dagger }}{\it{b}}_{{\mathrm{k{\prime} - q}}}^{\mathrm{\dagger }}{\it{b}}_{{\mathrm{k{\prime}}}}{\it{b}}_{\mathrm{k}}{\mathrm{ - 2}}{\it{V}}_{\mathrm{q}}^{{\mathrm{eh}}}{\mathrm{a}}_{{\mathrm{k + q}}}^{\mathrm{\dagger }}{\it{b}}_{{\mathrm{k{\prime} - q}}}^{\mathrm{\dagger }}{\it{b}}_{{\mathrm{k{\prime}}}}{\mathrm{a}}_{\mathrm{k}}} \right)}}$$

where ${\it{E}}_{\mathrm{k}}^{{\mathrm{e,h}}}$ are the single-particle electron and hole energies, **k** is the in-plane momentum, $a_{\mathrm{k}}^{\mathrm{\dagger }}\left( {a_{\mathrm{k}}} \right)$ and $b_{\mathrm{k}}^{\mathrm{\dagger }}\left( {b_{\mathrm{k}}} \right)$ are the creation (annihilation) operators for electrons in the conduction and holes in the valence bands, respectively, $V_{\mathrm{q}}^{{\mathrm{ee}}} = V_{\mathrm{q}}^{{\mathrm{hh}}} = \frac{{e^2}}{{2\varepsilon \left| {\mathrm{q}} \right|}}{\mathrm{,}}$and $V_{\mathrm{q}}^{{\mathrm{eh}}} = V_{\mathrm{q}}^{{\mathrm{hh}}} = \frac{{e^2}}{{2\varepsilon {\mathrm{q}}}}{\mathrm{e}}^{ - {\mathrm{q}} \cdot {\mathrm{d}}}{\mathrm{,}}$ where *ε* is the dielectric constant.

**Fig. 2:**
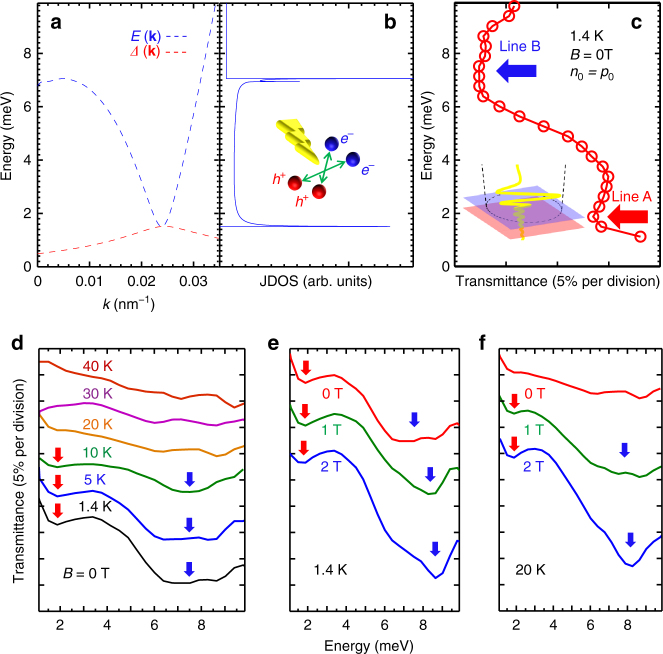
Pair-breaking excitation spectra of excitonic insulator. **a** Gap function *Δ*(**k**) (red dashed line) and the pair-breaking energy *E*(**k**) (blue dashed line) of the exciton as a function of *k* for *V*_b_ = 0 V. **b** Joint density of states as a function of energy. Inset figure: pair-breaking induced by THz light (Blue dots: electrons. Red dots: holes. Yellow arrow: THz light). **c** Transmission spectrum at the CNP at 1.4 K and 0 T for *V*_b_ = 0 V. In the inset figure, the purple layer represents the InAs quantum-well, whereas the red layer represents the GaSb quantum-well. The yellow wave indicates the THz light, and the dashed black circle marks the lateral extent of the focused THz beam at the sample position. The gate and contacts are not drawn here. **d** Transmittance spectra at various temperatures at 0 T. **e**, **f** Transmittance spectra at different magnetic fields at 1.4 and 20 K, respectively. The spectra are vertically offset for clarity. The measurement uncertainty in THz energy is ±0.2 meV

To solve this many-body interaction problem, we used a mean-field treatment^3,7–9^ to calculate the exciton dispersion, *E*(**k**), and the gap function *Δ*(**k**) (Fig. 2a). Similarly to the case of Cooper pairs, the e–h Coulomb correlations in the EI phase lead to an unstable Fermi surface, spontaneously opening a BCS-like gap (EI gap) *Δ*_max_ near the CNP reached by the Fermi level; this is the gap probed by transport measurements. On the other hand, as a ground state, the EI state absorbs incident photons, with individual electrons and holes in the final state. *E*(**k**) is the pair-breaking excitation spectrum: the energy cost of taking one loosely bound exciton out of the condensate and placing a pair of an individual electron and an individual hole in-plane-wave states of momentum **k**. Figure 2b shows the joint density of states (JDOS) we calculated, which predicts two singularity peaks: one peak near *E*_min_ ~ 1.5 meV (or ~18 K), and the other near *E*(**k**) ~ 7 meV (or ~80 K) with **k** ~ 0. There is fine structure in the 7 meV peak due to the existence of two nearby peaks. Note that the *E*(**k**) and *Δ*(**k**) curves in Fig. 2a are general EI characteristics, independent of material details^3,7–9^, making an EI distinguishable from an exciton BEC (or an exciton gas). For an exciton BEC/exciton gas, the two peaks merge at **k** = 0, corresponding to the exciton binding energy; furthermore, there is no spontaneous gap opening near the Fermi surface^7^. In the following, from both optics and transport perspectives, we present key findings that support these calculation results, thereby evidencing the existence of an EI in our system.

### Pair-breaking excitation measurement of excitonic insulator by terahertz spectroscopy

We performed low-temperature THz transmission spectroscopy experiments^16,23,24^ (see Methods) on a device covered by a 5 mm × 5 mm semi-transparent gate, in a frequency (energy) range of 0.25–2.4 THz (~1–10 meV). Note for THz experiment the wafer B is used which was prepared on a semi-insulating GaAs substrate. A transmittance spectrum when the system is at the CNP at 1.4 K is shown in Fig. 2c. Here, two absorption lines (or transmission dips) are present in the spectrum, line A at ~2 meV, and line B at 7.3 meV, consistent with what we expect from Fig. 2a and b. We interpret line A as coming from pair-breaking excitation near the Fermi level and line B corresponding to excitation near **k** = 0. Mention that what we are measuring is the joint density of states of the pair-breaking excitation process, which is characterized by the spectrum *E*(**k**), not a direct extraction of the gap. It should be noted that these features are observed only when *n*_0_ is low (~5.5 × 10^10^ cm^−2^); they disappear in an electron- or hole-dominating regime, or in a high *n*_0_ (~>10^11^ cm^−2^) case. In the case of line B, in addition to the fine structure in JDOS (Fig. 2b), inhomogeneous broadening due to random potential fluctuations would contribute to the linewidth^25^. Moreover disorder has stronger effect to low **k** states that have lower energy, contributing to a broader line. Also, random potential fluctuations are expected to enhance the exciton density at low **k** ^10^.

The interpretation of the lines as pair-breaking excitations across the BCS-like gap of an EI state is also supported by their temperature dependence (Fig. 2d). At 5 K, the unique two-line structure is still present, which cannot be explained by any single-particle gap in this system. The absorption amplitude of the lines, which directly reflects the JDOS of the excitations, significantly decreases when the temperature is raised to 10 K. At higher temperatures, the two-line structure is absent. A marked decrease of intensity of both lines occurs at a temperature (10 K) that is much less than the line energies (25 and 90 K, respectively). These results cannot be explained by a single-particle gap but can be interpreted as the transition from an EI state into a metallic state with a critical temperature *T*_c_ ~ 10 K, which also agrees with transport results as shown below.

When a magnetic field perpendicular to the QWs, *B*_⊥_, is applied at 1.4 K, the amplitudes of the absorption lines increase and line B slightly blue-shifts with the magnetic field, as shown in Fig. 2e. This *B*_⊥_-induced enhancement of the absorption lines is particularly marked at 20 K, where the lines are initially absent at 0 T, but the *B*_⊥_ causes them to re-emerge (Fig. 2f). The strengthened absorption under *B*_⊥_ is likely due to the fact that a *B*_⊥_ makes e–h pairs more tightly bound^7,26^, hence more stable against dissociation. Furthermore, it has theoretically been predicted that a magnetic field tends to stabilize the EI phase^27,28^, which is in stark contrast to BCS superconductors involving Cooper pairs (which are destroyed by a magnetic field).

Although the two lines (lines A and B) have different energies, they disappear at the same *T*_c_ and reappear simultaneously in *B*_⊥_. Such correlation can be quantitatively understood: both lines are associated with transitions from the EI state to distinct final states with different **k** values, as shown in the calculated spectrum (Fig. 2a). It not only confirms that the lines have the same origin but also provides optical spectroscopic evidence for the spontaneous formation of an EI gap through the Coulomb attraction between spatially separated electrons and holes.

### Excitonic insulator gap by Corbino measurement

**Fig. 3:**
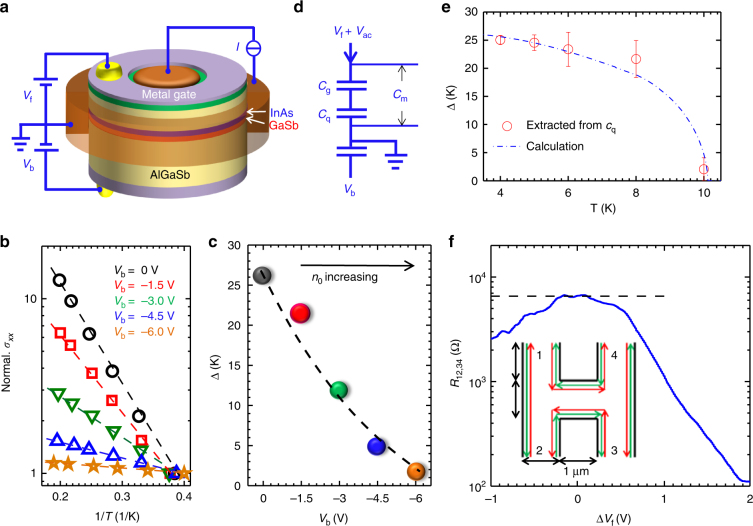
Excitonic insulator gap and topological edge states. **a** Sketch of the measurement setup. **b** Arrhenius plot of the conductance minimum for different back-gate bias voltages. The data can be fit by *σ*_*xx*_ ∝ exp(−*Δ*/2*k*_b_ *T*) to obtain *Δ*. Here the *σ*_*xx*_ is normalized by its value at 2.5 K. Dashed lines are guides to the eye. Black circles are for *V*_b_ = 0 V, red squares are for *V*_b_ = −1.5 V, green down-triangles are for *V*_b_ = −3 V, blue up-triangles are for *V*_b_ = −4.5 V, and yellow stars are for *V*_b_ = −6 V. **c** Measured gap energy *Δ* as a function of back-gate bias voltage. **d** Diagram for capacitance measurement. In addition to the dc *V*_f_ and *V*_b_, a small low frequency ac voltage was applied to the front-gate, and the capacitance between the front-gate and the quantum wells was measured. **e** Gap energy as a function of temperature for *V*_b_ = 0 V. At the charge-neutral point, the gap energies were extracted from *c*_q_. The dotted-dashed line is from the calculation. The error bars come from the uncertainties in *c*_q_ and the temperature. **f** Nonlocal measurement performed in a mesoscopic H-bar for *V*_b_ = 0 V at 0 T and 30 mK. The electrical current is passed through contacts 3 and 4, and the voltage is measured between contacts 1 and 2. The dashed line indicates the expected resistance value calculated from the Landauer–Büttiker formula. The edge current path is shown in the inset as red and green arrows

**Fig. 4:**
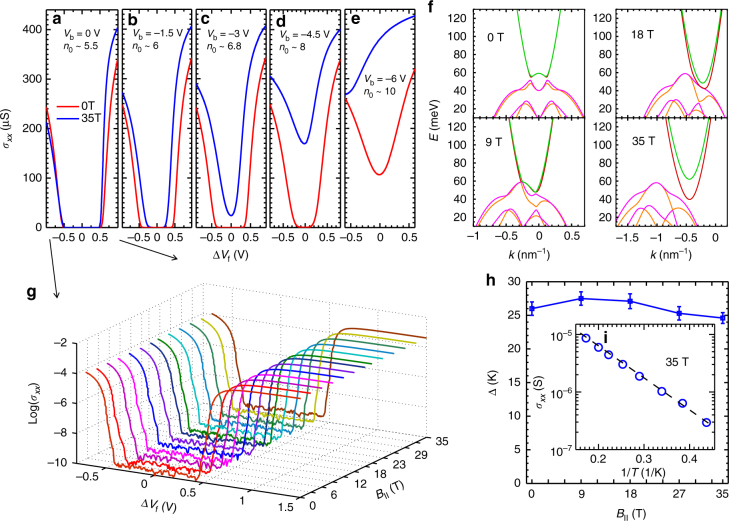
Measurement of density-dependent hybridization gap under in-plane magnetic fields. **a**–**e** Front-gate bias voltage dependence of *σ*_*xx*_ for a device at different back-gate bias voltages from 0 V to −6 V at 30 mK, with a decrement of 1.5 V. In each panel, the value of *n*_0_ is noted in units of 10^10^ cm^−2^. The blue (red) lines are for *B*_//_ = 35 T (0 T). **f** Energy dispersions calculated with an 8-band self-consistant model for tunneling electrons and holes with a typical inverted band at *B*_//_ = 0 T, 9 T, 18 T, and 35 T. **g** Front-gate bias voltage dependence of *σ*_*xx*_ for a Corbino device at zero back-gate at various *B*_//_ from 0 T to 35 T measured at 30 mK. A zero-conductance dip appears and persists up to 35 T. **h** Gap energy *Δ* vs. *B*_//_ at zero back-gate bias. The error bars come from the uncertainty in extraction of gap energy from Arrhenius plot. **i** Arrhenius plot of the dip conductance *σ*_*xx*_ at 35 T for two orders of magnitude. Dashed line is a guide to the eye

To access the gap function (order parameter) of the EI state, bulk conductance measurements (presented in Figs. 3 and 4) are necessary, and hence, we utilized a Corbino device (Fig. 3a) to exclusively measure bulk properties^19^. The red trace in Fig. 4a shows that the bulk conductivity drops fast with a conductance dip coming to zero at the CNP. Red lines from Fig. 4a (low *n*_0_ case, *V*_b_ = 0) to Fig. 4e (high *n*_0_ case, *V*_b_ = −6 V), the width of the zero-conductance region decreases; the *σ*_*xx*_ dip around the CNP is lifted from zero, indicating that the EI state is weakened by increasing *n*_0_. The opening of a hard gap in the low *n*_0_ case can be confirmed quantitatively via thermal activation measurements. The *σ*_*xx*_ at CNP vs. 1*/T* can be fit over one order of magnitude in conductivity with an Arrhenius function $\sigma_{xx} \propto$ exp(−*Δ*/2*k*_b_ *T*), with activation energy *Δ* ~ 2 meV (or  ~ 25 K) (black dashed line in Fig. 3b, also see Supplementary Fig. 3 and Supplementary Note 1). Our calculated gap energy (blue dashed line in Fig. 2a) agrees with this energy value well.

Next, we measured the gap as a function of *n*_0_. The data taken at the CNP follows the relation $\sigma_{xx}\propto$ exp(−*Δ*/2*k*_b_ *T*) well, yielding a set of *Δ* values as plotted in Fig. 3c. The gap energy diminishes steeply to nearly zero as *n*_0_ increases (Fig. 3c). Hence, the gap energy is strongly correlated with 1/*n*_0_, suggesting that a low *n*_0_ is crucial for the formation of an EI gap. These results can be understood as a consequence of weakened e–h binding through increased screening as the *n*_0_ increases.

### Temperature dependence of excitonic insulator gap by capacitance measurement

On the other hand, as the temperature increases, the EI state becomes unstable and the gap eventually closes. We studied the behavior of the gap as a function of temperature through capacitance spectroscopy experiments (Fig. 3d) (see Methods and Supplementary Fig. 4). *c*_m_ = 1/(1/*c*_g_ + 1/*c*_q_) is the capacitance measured per unit area, where *c*_g_ is the geometry capacitance per unit area, ${\it{c}}_{\mathrm{q}} = {\it{e}}^{\mathrm{2}}{\it{D}}$ is the quantum capacitance per unit area, and *D* is the density of states (DOS). We begin with an analysis of the quantum capacitance in different regimes. At *T*_c_ ~ 10 K, the gap vanishes and the *D* is dominated by electrons and holes; we take 1/*c*_q_ = 0 as a reference point. At *T *~ 0, the *D* is zero since it would take an energy cost of *Δ*(*T*) to excite a pair of electron and hole. At an intermediate temperature, *D* is proportional to (1/*k*_b_ *T*)exp(−*Δ*/2*k*_b_ *T*); measurement of *c*_q_ is yielding a semi-quantitative estimation of the gap function *Δ*(*T*) as shown in Fig. 3e. We can see that *Δ* starts decreasing at 5 K and finally diminishes at 10 K. This behavior can be well described by the gap function predicted for EI (see Supplementary Fig. 5 and Supplementary Note 2), but is inconsistent with alternative interpretations such as thermal excitations over a single-particle band gap.

### Nonlocal measurement of edge states in topological excitonic insulator

In the low *n*_0_ regime comparable to the case here, the quantization plateau of helical edge states has been previously observed^19^ and taken as the evidence of the QSH effect. Moreover, the plateaus were found to be quite robust under the variance of external parameters such as in-plane magnetic field or temperature. Such a robustness of helical edge states cannot be explained by existing single-particle theory concerning 2D topological insulators. Pikulin and Hyart subsequently proposed^20^ the emergence of an unconventional EI ground state in the inverted InAs/GaSb QWs, providing a plausible explanation for these experimental observations. In their model, the interplay between an excitonic ground state and interlayer tunneling, which is naturally existing in the present InAs/GaSb structure (i.e., without a tunneling barrier between the two layers) can lead to a *p-*wave EI with topologically protected edge states^19^. The present work has provided convincing experimental evidences for the existence of EI gap in this system when it is tuned into low densities.

To explore the nontrivial topological properties of the condensate, we performed nonlocal transport measurements in a mesoscopic H-bar device at low *n*_0_. According to the Landauer–Büttiker formula (see Supplementary Note 3), for helical edge transport, *R*_12,34_ = *V*_12_ */I*_43_ should measure quantized resistance of *h/*4*e* ^2^ ~ 6.45 kΩ. As shown in Fig. 3f, we indeed observed a quantized plateau close to this value, which confirms that helical edge transport^29^ is indeed realized, whereas the bulk is insulating (also see Supplementary Notes 4 and 5, and Supplementary Figs. 6 and 7). Together with the above-presented optical and transport evidence, these results suggest that an EI spontaneously emerges in the bulk of InAs/GaSb QWs with helical edge modes propagating along the perimeters, consistent with the observed unusual QSH properties in InAs/GaSb^19^ within the picture of the proposed topological EI^20^.

### Measurement of density-dependent hybridization gap under in-plane magnetic fields

In InAs/GaSb QWs without a middle barrier layer, conduction band/valence band hybridization is a potential mechanism for the opening of a bulk gap. However, its contribution to the gap can be distinguished by the application of an in-plane magnetic field *B*_//_^30^. Within the single-particle picture, electrons and holes with the same momentum tunnel between QWs, forming a hybridization gap. An applied *B*_//_ induces a relative shift of band dispersions by the amount *eB*_//_ *d*/*h*. Consequently, the inter-well tunneling is suppressed due to momentum mismatch, or in other words, *B*_//_ creates an effective barrier for tunneling. As shown in our 8-band self-consistent calculations (Fig. 4f) in the tunneling regime (see Supplementary Notes 6 and 7, and Supplementary Fig. 8), as *B*_//_ increases beyond 18 T, the two bands will separate in **k** space, and the system becomes a semimetal. Here, we applied a *B*_//_ of 35 T to the Corbino device (also see Supplementary Note 8 and Supplementary Fig. 9) and took the conductance increment *(σ*_*xx*_(35 T) − *σ*_*xx*_(0 T)) at the CNP as a qualitative measure of the contribution from single-particle hybridization to the gap. For *V*_b_ = −6 V (the highest *n*_0_ ~ 1 × 10^11^ cm^−2^), *σ*_*xx*_(35 T) increases from the *B* = 0 value by four times and the dip vanish at 35 T, indicating that the hybridization effect is dominant. As *n*_0_ decreases, *σ*_*xx*_(35 T) still deviates from *σ*_*xx*_(0 T) but has a dip at the CNP, showing that the hybridization has a weaker role and the EI gap gradually appears. At *V*_b_ = 0 V (lowest *n*_0_ ~5.5 × 10^10^ cm^−2^), *σ*_*xx*_ is characteristically the same as that at *B* = 0, which is further demonstrated by a plot of *σ*_*xx*_ at various *B*_//_ with a broad zero-conductance region seen from *B*_//_ = 0 to 35 T (Fig. 4g). In the presence of a *B*_//_, *σ*_xx_ markedly depends on *n*_0_, demonstrating that the EI phase is more stable at a lower *n*_0_. At the lowest *n*_0_, the gap remains open in spite of strong in-plane magnetic fields, confirmed by activation measurements under different *B*_//_ up to 35 T in Fig. 4h and i. The observed systematic responses of the gap conductance to the *B*_//_ support the notion that the EI phase, not the hybridization effect, is responsible for the appearance of the gap in such a low density, equilibrium electron–hole gas.

## Discussion

The realization of an excitonic insulator state in our InAs/GaSb system paves the way for further studying many-exciton physics in great detail as well as depth (e.g., BCS-BEC crossover, and BEC exciton) in an equilibrium electron–hole system without optical pumping. Due to the weak binding nature of the EI and a lack of middle barrier, counter-flow experiments on the current system would not be as effective as in the BEC case^12^. With an additional AlGaSb barrier between the electron and hole layers, counter-flow studies would be enabled to explore BCS to BEC crossover. Moreover, the inverted band structure of the current system brings in topological nature to the EI state, which will allow one to study 2D interacting topological insulators in a highly controllable manner. For example, with a thin AlGaSb middle barrier or utilizing strained-layer InAs/GaInSb QWs, the symmetry of the order parameter can be controlled by tuning the interplay of interlayer interactions and tunneling.

## Methods

### Characterization and transport measurements

Al_0.8_Ga_0.2_Sb–InAs/GaSb–Al_0.8_Ga_0.2_Sb wafers were prepared by molecular beam epitaxy (MBE) on (001) substrate with a 1 μm thick buffer layer, with the following nominal parameters: Wafer A, on N + GaAs substrate, with 12.5 nm InAs/10 nm GaSb QWs; Wafer B, on SI- GaAs substrate, with 11.5 nm InAs/8 nm GaSb QWs; Wafer C, on *n*-GaSb substrate, with 11 nm InAs/7 nm GaSb QWs. In Wafer A and B, the interface between the GaSb and InAs QWs was doped with a dilute sheet of Si with a concentration of ~1 × 10^11^cm^−2^, whereas there was no doping in Wafer C.

Characterization of wafer A can be found in ref. ^19^. In wafer A, Supplementary Fig. 2a and b shows *B/eR*_*xy*_ vs. *ΔV*_f_ for a Hall bar device at *V*_b_ = −6 V and 0 V, respectively; *B* is the perpendicular magnetic field and *ΔV*_f_ is the front-gate bias increment from the CNP. For *V*_b_ = −6 V, at the high electron density (regime I), *B/eR*_*xy*_ is consistent with the density obtained from Subinikov de Haas oscillations, linear with *V*_f_. As the top of the hole band is reached by the Fermi level, holes are introduced and two-carrier transport dominates, with *R*_*xy*_ traces divergent and the system reaching the e–h hybridized regime (regime II). Owing to the absence of a hard gap, *B/eR*_*xy*_ is still dominated by electron–hole residual carriers even in the hybridization gap (regime III). As the Fermi level is below the bottom of the electron band, holes dominate and *R*_*xy*_ becomes linear again (regime IV). For *V*_b_ = 0 V, where less holes are introduced by *V*_b_, as the CNP is approached, similarly the system goes from regime i to regime II. However, within regime II, a plateau-like feature is observed, indicating the formation of an EI gap (regime V).

In Wafer C, lattice matched epitaxial layers were grown on a GaSb substrate, which should result in enhanced carrier mobility. The carrier mobility of Wafer C measured at 300 mK indeed showed significant improvement. The typical electron mobility was 90,000 cm^2^ V^−1^ s^−1^ for a density of 5 × 10^11^ cm^−2^, and the hole mobility was about one order of magnitude lower at similar densities. Magneto-transport experiments showed well-resolved quantum oscillations, as shown in Supplementary Fig. 1a. In a perpendicular magnetic field, the device can be tuned by the front-gate from the electron dominating regime to the hole-dominating regime in an asymmetrical Hall bar, as shown in Supplementary Fig. 1b. As the electron density decreases, holes emerge, which bends the trace. When the hole and electron densities become comparable, the trace drops again with an EI gap emerging and helical edges dominating. The equilibrium density *n*_o_ was ~5 × 10^10^ cm^−2^.

The *V*_f_ dependence of the conductance in a Corbino device C2 (made from Wafer C) is shown in Supplementary Fig. 1c. Similar to Wafer A, there is a conductance dip at the CNP with the resistivity equal to ~1 MΩ/square. As we apply *B*_//_ up to 35 T, the dip conductance stays nearly the same, which confirms that this gap is not from hybridization, as shown in Supplementary Fig. 1d. Furthermore, the constant dip conductance also shows that the gap does not close between 0 T and 35 T. These findings are very similar to those from Wafer A, suggesting that Si doping is not essential for the emergence of the EI gap.

### Terahertz measurements

In optical spectroscopy experiments, we measured the transmission of a terahertz (THz) beam through the device made from Wafer B, which was prepared on a semi-insulating GaAs substrate. The device had a semi-transparent front-gate, and the gated area was 5 mm × 5 mm for maximizing the THz transmission. Through the semi-transparent front-gate, we modulated the system between the CNP and the low mobility electron regime. The electron regime we selected had low mobility and low density, so it had no feature in the 0.25–2.4 THz frequency range, working as a good reference for the exciton-induced transmission. The transmittance was defined as the intensity of the THz beam transmitted through the sample normalized by the reference signal.

### Capacitance–voltage measurements

We performed capacitance–voltage (CV) measurements^30^ under different *V*_b_ in Corbino devices C1 (Wafer A) and C2 (Wafer C) (Supplementary Fig. 4a). In addition to the dc *V*_f_ and *V*_b_, a small ac (*f* = 100 Hz) voltage was applied to the front-gate, and the capacitance between the front-gate and the QWs was measured. In CV measurements^30^, the geometry capacitance *c*_q_ and quantum capacitance *c*_q_ = *e* ^2^ *D* contribute to the measured capacitance per unit area *c*_m_ as *c*_m_ = 1/(1/*c*_g_ + 1/*c*_q_), where *D* is the density of states (DOS). In the electron or hole-dominating regime, *c*_g_ is much smaller than the quantum capacitance, so *c*_g_ dominates in *c*_m_. When the DOS decreases and quantum capacitance is smaller than *c*_g_, *c*_m_ drops and the quantum capacitance dominates.

The out-of-phase signal was one order of magnitude less than the in-phase signal. In this case, for devices from both Wafers A and C, the observed capacitance drop near the CNP represented the reduced DOS. For *V*_b_ = −6 V, as illustrated in the blue trace of Supplementary Fig. 4a, the capacitance near the CNP was nearly constant, agreeing with previous CV results^30^ about the hybridized gap (the tunneling is too weak to form a hard gap). The DOS in this gap can be treated as the mixture of electron’s and hole’s. For *V*_b_ = 0 V, the capacitance at the CNP drops to 10% of *c*_g_, which indicates that the hard gap (EI gap) forms with a reduced DOS. CV measurements performed on Corbino device C2 with *V*_b_ = 0 V also showed that there is a capacitance drop near the CNP (Supplementary Fig. 4b), confirming that the EI gap exists and the gap is not from Si doping. It should be mentioned that in Wafer C there was no doping and thus, Anderson localization (AL) was suppressed. Moreover, AL could not reduce the DOS. Therefore, the CV results confirm that the hard gap does not originate from AL. A large capacitance drop shows that the co-existing electrons and holes can form neutral particles (reduced DOS) with the formation of the EI gap.

Next we increased the temperature. Supplementary Fig. 4c shows CV traces at *V*_b_ = 0 V for different temperatures. We find that the EI gap starts collapsing at 6 K and disappears at 10 K. This confirms that the EI gap dominates in the low-temperature regime, and also provides the temperature window for thermal activation energy measurements.

### Determination of equilibrium density of electron and hole

Ideally, the electrons are tuned by the front-gate, whereas the holes are tuned by the back-gate. Owing to the imperfect screening from the electron layer, the front-gate could simultaneously modulate the electrons and holes when holes appear.

Supplementary Fig. 12a shows that, for Δ*V*_f_ sweeping from 2 V, the electron density is high and linear with Δ*V*_f_, so we can extract the electron density increment per Δ*V*_f_. Supplementary Fig. 12b shows the electron density rate under different *V*_b_. The rate is constant for |*V*_b_| < 3 V. When |*V*_b_| increases and holes appear, the rate decreases until *V*_b_ = −7.5 V, where the rate saturates.

From CV measurements, we know that the capacitance stays constant until the EI gap appears. The capacitance per unit area *c*_m_ means the absolute density (|*n*| + |*p*|) increment per Δ*V*_f_. So we can integrate the capacitance over Δ*V*_f_ to obtain the absolute density, as shown in Supplementary Fig. 12a. It should be mentioned that, for the high electron density, the hole density can be neglected, so the absolute density agrees with the electron density. The absolute density decreases with a constant rate until it gets to nearly zero, as shown in the blue dashed line, where the CNP is approached. In this case, we can obtain the voltage where the CNP is reached (vertical dashed black line).

In the magneto-transport trace (red dotted line in Supplementary Fig. 12a), as the holes emerge, the trace bends up and it is not straight forward to extract the electron density. However it is reasonable to make an approximation that, when holes appear, the electrons change in the saturated rate (green dashed line) until the CNP is reached, as illustrated in Supplementary Fig. 12a. This way we can estimate the equilibrium density *n*_0_ (marked by the circle). The density uncertainty is 5 × 10^9^ cm^−2^. Similarly, we obtain *n*_0_ under different *V*_b_, as shown in Supplementary Fig. 13.

### Data availability

The authors declare that the data supporting the findings of this study are available within the paper and its Supplementation Information files or from the corresponding author upon reasonable request.

## Electronic supplementary material

Supplementary Information
