# Supplementary material for: Evidence for a topological excitonic insulator in InAs/GaSb bilayers
Source: Nat Commun. 2017 Dec 7;8:1971. doi: 10.1038/s41467-017-01988-1 (PMC5719361; doi:10.1038/s41467-017-01988-1)
Supplement: Supplementary file 1 — Supplementary Information [file 41467_2017_1988_MOESM1_ESM.pdf]

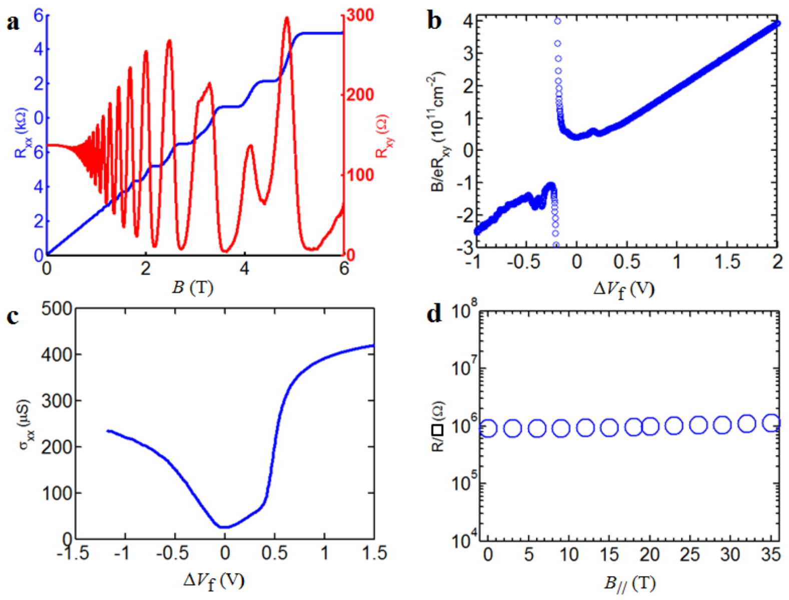

Supplementary Figure 1. Characterization and transport measurement of wafer C. a shows magneto-resistance and Hall resistance traces at 300mK. **b,** B/eR_{xy} –ΔV_f curves of the asymmetric 50μm × 50μm Hallbar device at 300mK. **c,** ΔV_f dependence of the conductance for Corbino device C2 at 30mK. **d,** The resistance per square in the excitonic insulator gap under $B_{//}$ up to 35T in 2 or 3T increments.

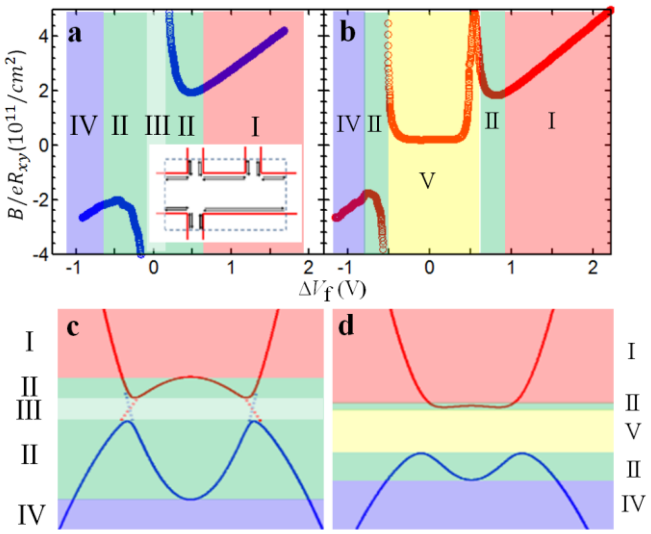

Supplementary Figure 2. Magneto-transport in an asymmetric Hall bar device. a and **b,** B/eR_{xy} versus ΔV_f traces of the asymmetric 50μm × 50μm Hall bar for V_b= -6 and 0V, respectively; the data were taken at 300mK with a 1T perpendicular magnetic field. The inset in **a** is a schematic of the asymmetric Hall bar, and the region in the dashed box is covered by the front gate. **c** and **d** show band alignments corresponding to **a** and **b**, respectively. The red regime I is the electron dominating regime. The blue regime IV is the hole dominating regime. The green regime II is the electron-hole coexisting regime. The light green regime III is the soft gap. The dotted line means residual electron and hole filling in hybridization gap so in **a** there is no hard gap observed. The gold regime V is the hard gap (excitonic insulator gap).

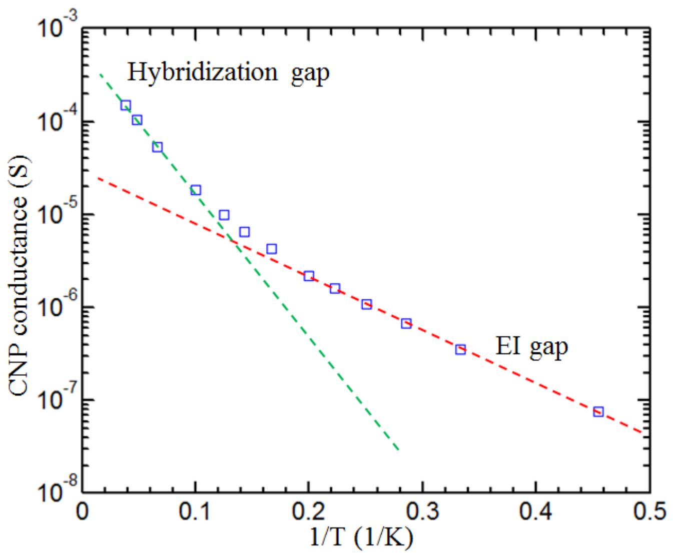

Supplementary Figure 3: Arrhenius plot of the conductance minimum at V_b = 0 V. The blue line indicates the hybridization gap while the red line indicates the excitonic insulator gap.

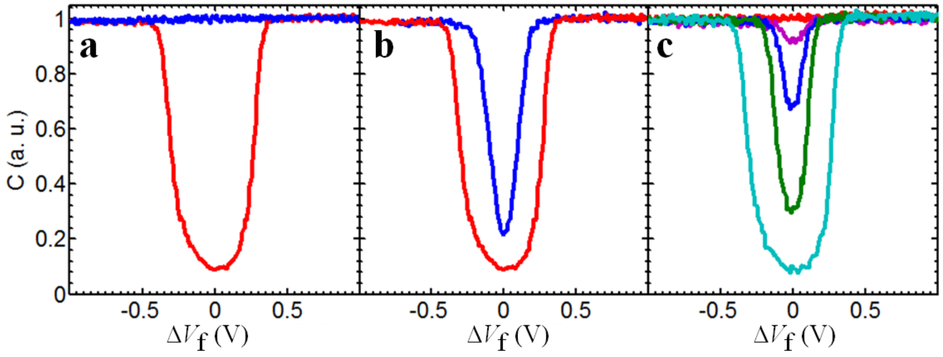

Supplementary Figure 4. Capacitance-voltage measurement. a, Capacitance-voltage curves with V_b=0V (red line) and -6V (blue line). At 300mK, a low frequency (100Hz) ac voltage is delivered to the frontgate with quantum wells grounded, and the capacitance between the front gate and quantum wells can be measured. **b** shows capacitance-voltage curves with V_b=0V from wafer A (red line) and wafer C (blue line), respectively. In both cases, large capacitance drops exist. **c,** capacitance-voltage curves under different temperatures (0.3K, 2K, 4K, 6K and 10K). Curves are taken in C1 with V_b=0. The observed excitonic insulator gap disappears for the temperature up to 10K.

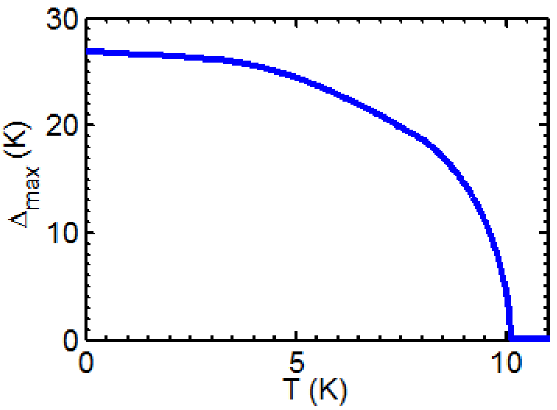

Supplementary Figure 5. The maximum of Δ(k) as a function of temperature for excitonic insulator in inverted InAs/GaSb quantum wells.

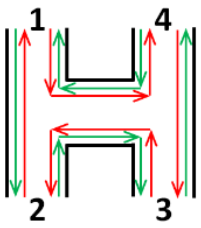

Supplementary Figure 6. Four-terminal H bar device with quantum spin hall edge states. The edge state in one spin direction follows the red line, while the other edge state in different spin direction goes through the green line.

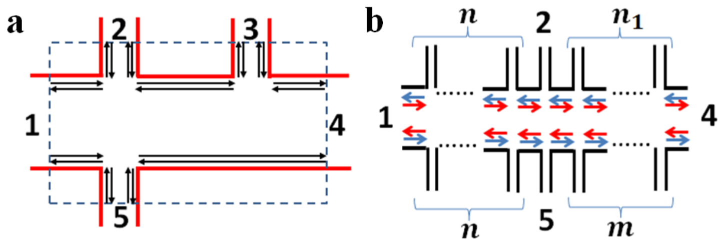

Supplementary Figure 7. Edge states in an unsymmetrical macro-Hallbar device. **a,** Schematic layout of unsymmetrical macro-Hallbar device. Blue dashed square is the front gate covered region. **b,** Illustration for edge transport after inserting virtual phase-breaking scatters. There are n scatters between contacts 1 and 2(5), n_1 scatters between contacts 2 and 4, and m scatters between contacts 4 and 5.

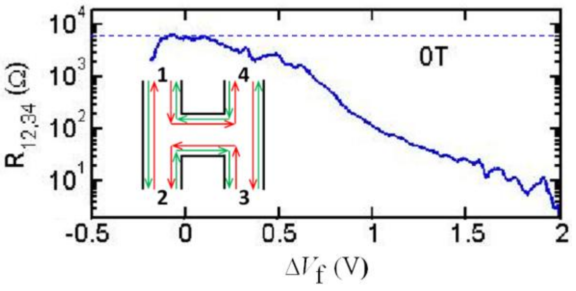

Supplementary Figure 8. Nonlocal measurement performed on a meso-H bar from wafer C at 0T at 30mK. The dotted lines correspond to the expected resistance value based on Landauer-Büttiker formula. In the inset, the edge state in one spin direction follows the red line, while the other edge state in different spin direction goes through the green line.

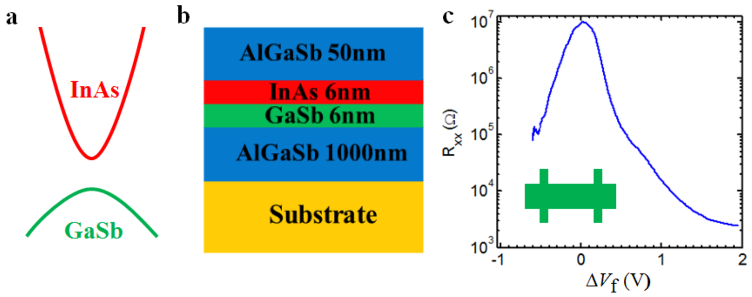

Supplementary Figure 9. Edge measurement on noninverted InAs/GaSb quantum wells. **a.** Band inversion of noninverted InAs/GaSb quantum wells. **b.** Detailed layer structure of the quantum wells. **c.** R_{xx} vs ΔV_f in a 10μm × 20μm Hallbar from the noninverted quantum wells at 300mK and 0T.

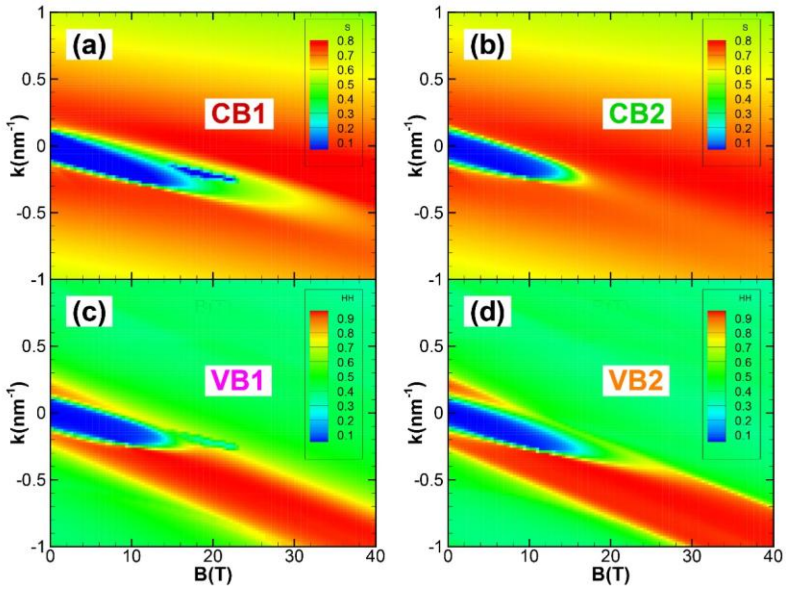

Supplementary Figure 10. The contour plots of subband components. The contour plots of subband components with magnetic field and k_x for (**a**) the first conduction band (CB1), (**b**) the second conduction band (CB2), (**c**) the first valance band (VB1), and (**d**) the second valance band (VB2), respectively.

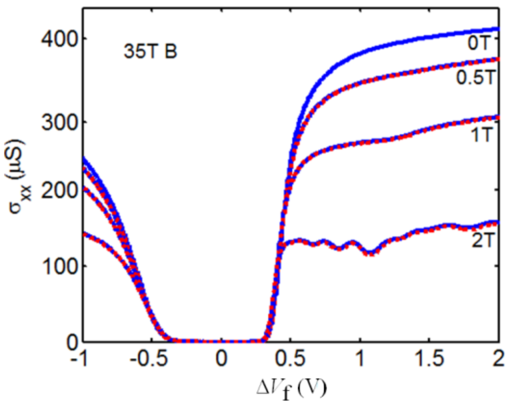

Supplementary Figure 11. Magneto-conductance of a Corbino device under small perpendicular magnetic fields. The total magnetic field is 35T and the temperature is 30mK. By rotating the device, the device plain has a deviation angle with the magnetic field so that there are small perpendicular magnetic fields (0T- ±2T). The blue solid lines are for positive perpendicular magnetic fields while the red dotted lines are for negative perpendicular magnetic fields.

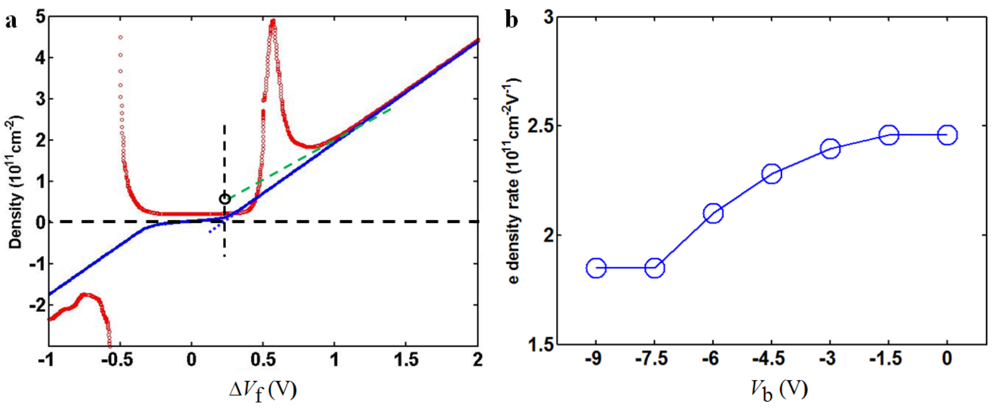

Supplementary Figure 12. Determination of the charge neutral point density. a, Illustration for the equilibrium density at the charge neutral point. The red dotted line is for magneto transport of unsymmetrical macro-Hallbar. In the linear part of the trace near 2V, the trace represents the electron density. The blue line is for the capacitance integration over ΔV_f which represents the absolute density. The green dashed line is for the electron density changing in the saturated rate. n_o could be read from the crossing point of vertical dashed black line and green dashed line. **b,** Electron density increment per ΔV_f in high electron density regime for different V_b.

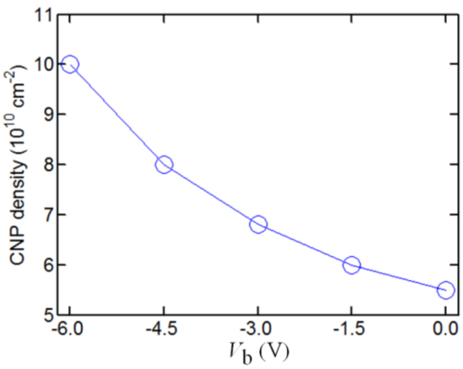

Supplementary Figure 13. Charge neutral point density n_o as a function of V_b. The density uncertainty is 5×10^9 cm^{-2}.

Supplementary Table 1. The Kane parameters[1] used in our calculation.

	InAs	GaSb	AlSb
E_g (eV)	0.417	0.812	2.386
E_v (eV)	-0.417	0.143	-0.237
Δ (eV)	0.39	0.76	0.676
A_c (eV nm^2)	-0.26	0.09	-0.06
γ_1	2.01	4.16	2.04
γ_2	-0.49	8.18	-0.38
γ_3	0.21	1.38	0.40
ε	14.55	15.69	14.4

Supplementary Note 1: Activation measurements in an extended temperature regime

As shown in Supplementary Figure 3, in the low temperature regime well below the T_c of the condensation, the CNP resistance follows the red line, in accordance of the EI gap. As the temperature increases and is above 10 K, the CNP resistance follows the green line, indicating the dominance of a hybridized state with a soft gap, as reported in several studies on this system[2,3,4].

Supplementary Note 2: Theory of the temperature dependence of the order parameter

The Hamiltonian in the second quantization form can be expressed as

$$\boldsymbol{H}=\sum_{\mathbf{k}}([\epsilon_a(\mathbf{k})-\mu]a_{\mathbf{k}}^{\dagger}a_{\mathbf{k}}+[\epsilon_b(\mathbf{k})$$

$$-\mu]b_{\mathbf{k}}^{\dagger}b_{\mathbf{k}})+\frac{1}{2}\sum_{\mathbf{k},\mathbf{k}',\mathbf{q}}[V_{aa}(\mathbf{q})a_{\mathbf{k+q}}^{\dagger}a_{\mathbf{k}'-\mathbf{q}}^{\dagger}a_{\mathbf{k}'}a_{\mathbf{k}}+V_{bb}(\mathbf{q})b_{\mathbf{k+q}}^{\dagger}b_{\mathbf{k}'-\mathbf{q}}^{\dagger}b_{\mathbf{k}'}b_{\mathbf{k}}\text{-}2V_{ab}(\mathbf{q})a_{\mathbf{k+q}}^{\dagger}b_{\mathbf{k}'-\mathbf{q}}^{\dagger}b_{\mathbf{k}'}a_{\mathbf{k}}] \quad (1)$$

where $a_{\mathbf{k}}, a_{\mathbf{k}}^{\dagger}$ and $b_{\mathbf{k}}, b_{\mathbf{k}}^{\dagger}$ are the destruction and creation operators of electrons in the conduction band and holes in the valance band, respectively, $V(\mathbf{q})$ is the interaction between carriers, and μ is the chemical potential. Following Ref. 5, we first use the mean field approximation and then diagonalize the pair Hamiltonian by introducing a Bogoliubov transformation of H, which results in a numerical (not operator) term in the Hamiltonian. Minimizing this term with respect to the transformation constant leads to the generalized BCS equations

$$\Delta(\mathbf{k})=\sum_{k'}V_{\mathbf{k}-\mathbf{k}'}^{ab}\frac{\Delta(\mathbf{k}')}{E(\mathbf{k}')}[1-f\left(\frac{E\left(\mathbf{k}'\right)-\eta\left(\mathbf{k}'\right)}{2}\right)-f\left(\frac{E\left(\mathbf{k}'\right)+\eta\left(\mathbf{k}'\right)}{2}\right)]$$

$$\xi(k)=\epsilon_{\mathrm{P}}(\mathbf{k})-\mu-\sum_{k'}V_{\mathbf{k}-\mathbf{k}'}^{aa}\ [1-\frac{\xi\left(\mathbf{k}'\right)}{E\left(\mathbf{k}'\right)}][1-f\left(\frac{E\left(\mathbf{k}'\right)-\eta\left(\mathbf{k}'\right)}{2}\right)$$

$$-f\left(\frac{E\left(\mathbf{k}'\right)+\eta\left(\mathbf{k}'\right)}{2}\right)]$$

$$\eta(\mathbf{k}) = \epsilon_{\mathrm{M}}(\mathbf{k}) - \mu - \sum_{\mathbf{k}'} V^{ab}_{k-k'} \left[1 - f\left(\frac{E\left(\mathbf{k}'\right) - \eta\left(\mathbf{k}'\right)}{2}\right) + f\left(\frac{E\left(\mathbf{k}'\right) + \eta\left(\mathbf{k}'\right)}{2}\right)\right] \qquad (2)$$

and $E^2(\mathbf{k}) = \Delta^2(\mathbf{k}) + \xi^2(\mathbf{k})$. The $\epsilon_{\mathrm{P}}(k)$ and $\epsilon_{\mathrm{M}}(k)$ can be expressed as $\epsilon_P(\mathbf{k}) = \epsilon_a(\mathbf{k}) + \epsilon_b(\mathbf{k})$ and $\epsilon_M(\mathbf{k}) = \epsilon_a(\mathbf{k}) - \epsilon_b(\mathbf{k})$, respectively. The function $f(x) = (e^x + 1)^{-1}$ is the Fermi distribution function. The above equations are coupled equations that must be solved self-consistently. The numerical result, the maximum of $\Delta(\mathbf{k})$ as a function of temperature, is shown in Supplementary Figure 5.

Supplementary Note 3: Helical edge transport

The Landauer-Büttiker (LB) formula describes helical edge transport in multi-terminal devices in terms of transmission functions. The relationship between terminal current and voltages is given by[6]

$$I_i = e^2/h \sum_j T_{ji}V_i - T_{ij}V_j \qquad (3)$$

where i and j are contact labels, and T_{ij} is the transmission from contact i to j. T_{ij} = 1 if i and j are neighbors; otherwise, T_{ij} =0.

For helical edges states in a four-terminal H bar device (Supplementary Figure 6), the edge channel goes from one contact to a neighboring contact. When the current goes through Contact 1 to 2, according to the LB formula, we can measure the voltage between Contacts 3 and 4 and get V_4-V_3=$I_1 h/(4e^2)$ and $G_{12,43}$=$4e^2/h$. On the other hand, the current going through Contacts 2 and 3 is $I_1/4$. Therefore, we obtain quantum resistance $R_{23,23}$=h/e^2 which connects neighboring contacts.

When the size of the device is longer than the phase coherence length, phase-breaking scattering occurs within the edge states[2, 3]. We can estimate the conductance by inserting several virtual contacts between real contacts due to the lack of phase coherence. Edge states between virtual contacts are coherent and connected with quantum resistance. So, the problem of a macro-size edge becomes that of a meso-size edge. For an unsymmetrical macro-Hall bar, as shown in Supplementary Figure 7a, the current goes through Contacts 1

and 4. There are two contacts in one side of the Hall bar while there is one contact in the other side. The Hall signal can be measured between Contacts 2 and 5. By inserting virtual contacts (Supplementary Figure 7b), the resistance between Contacts 2 and 5 becomes $R_{14,52}=h/e^2(n_1\text{-}m)(n+1)/(n_1+m+2n+4)$and we get the coherence length of ~5 μm, which agrees with a previous result[2]. Therefore, when the system is dominated by helical edge transport, at the CNP there is a high resistance in the Hall signal resulting from the geometry of the device and the coherence length.

Supplementary Note 4: Nonlocal measurements in a mesoscopic H bar device from wafer C

We performed nonlocal measurements in a mesoscopic H-bar device made from wafer C. According to the Landauer-Büttiker formula, $R_{12,34}$ should measure a quantized resistance of $h/4e^2$ ~ 6.45 kΩ. We indeed observed a quantized plateau close to this value within the EI gap regime, as shown in Supplementary Figure 8.

Supplementary Note 5: Transport in noninverted InAs/GaSb QWs

In a noninverted InAs/GaSb QW device (Supplementary Figure 9a), both the InAs and GaSb layers had a thickness of 6 nm (Supplementary Figure 9b). This QW thickness would result into a noninverted band with a normal semiconducting gap. Then a 10 μm × 20 μm Hall bar was fabricated in the same way as those in Fig. 3f and others, and measured as shown in Supplementary Figure 9c. Similar to the previous results from other groups[3], the resistance at the CNP reached 10 MΩ. Considering the limit of our lock-in amplifier, we know that the actual resistance would be much larger. This result shows a sharp contrast to those in the inverted band[2], confirming that there is no trivial edges formed in our InAs/GaSb devices due to artificial reasons such as the fabrication processing.

Supplementary Note 6: Details of self-consistent calculations based on the eight-band k·p theory

First, we give the details of 8-band self-consistent band structure calculations of InAs/GaSb quantum wells (the calculation details can also be found in Ref. [7]). The electronic structure of narrow gap semiconductor heterostructures can be well described by the eight-band **k·p** Kane model.

By choosing the following set of basic functions

$\Phi_1=|1/2,1/2>=|S\uparrow>$,

$\Phi_2=|1/2,-1/2>=|S\downarrow>$,

$\Phi_3=|3/2,3/2>=|(X+iY)\uparrow>/\sqrt{2}$,

$\Phi_4=|3/2,1/2>=i|(X+iY)\downarrow-2Z\uparrow>/\sqrt{6}$,

$\Phi_5=|3/2,-1/2>=|(X-iY)\uparrow+2Z\downarrow>/\sqrt{6}$,

$\Phi_6=|3/2,-3/2>=i|(X-iY)\downarrow>/\sqrt{2}$,

$\Phi_7=|1/2,1/2>=|(X+iY)\downarrow+Z\uparrow>/\sqrt{3}$,

$\Phi_8=|1/2,-1/2>=-i|(X-iY)\uparrow-Z\downarrow>/\sqrt{3}$,

where $|j,m>$ is the basis function, j is total angular momentum of electronic states and m is the z component of angular momentum. $|X>$, $|Y>$, and $|Z>$ are the atomic p orbital wave

functions and |S> is the atomic s orbital wave function. Kane Hamiltonian for the zinc-blende crystal near the Briouin zone center Γ point is written as

$$H_0(\mathbf{k}) = \begin{bmatrix} A & 0 & i\sqrt{3}V^\dagger & \sqrt{2}U & iV & 0 & iU & \sqrt{2}V \\ 0 & A & 0 & -V^\dagger & i\sqrt{2}U & -\sqrt{3}V & i\sqrt{2}V^\dagger & -U \\ -i\sqrt{3}V & 0 & -(P+Q) & L & M & 0 & iL/\sqrt{2} & -i\sqrt{2}M \\ \sqrt{2}U & -V & L^\dagger & -(P-Q) & 0 & M & i\sqrt{2}Q & i\sqrt{3}L/\sqrt{2} \\ -iV^\dagger & -i\sqrt{2}U & M^\dagger & 0 & -(P-Q) & -L & -i\sqrt{3}L^\dagger/\sqrt{2} & i\sqrt{2}Q \\ 0 & -\sqrt{3}V^\dagger & 0 & M^\dagger & -L^\dagger & -(P+Q) & -i\sqrt{2}M^\dagger & -iL^\dagger/\sqrt{2} \\ -iU & -i\sqrt{2}V & -iL^\dagger/\sqrt{2} & -i\sqrt{2}Q & i\sqrt{3}L/\sqrt{2} & i\sqrt{2}M & -P-\Delta & 0 \\ \sqrt{2}V^\dagger & -U & i\sqrt{2}M^\dagger & -i\sqrt{3}L^\dagger/\sqrt{2} & -i\sqrt{2}Q & iL/\sqrt{2} & 0 & -P-\Delta \end{bmatrix}$$

where $A = E_\mathrm{v} + E_\mathrm{g} + kA_\mathrm{c}k$, $P = -E_\mathrm{v} + \hbar^2\mathbf{k}\gamma_1\mathbf{k}/(2m_0)$, $Q = \hbar^2\left(k_x\gamma_2 k_x + k_y\gamma_2 k_\mathrm{y} - 2k_z\gamma_2 k_z\right)/(2m_0)$, $L = i\sqrt{3}\hbar^2\{k_-\gamma_3 k_z\}/m_0$, $M = -\sqrt{3}\hbar^2\left[k_x\gamma_2 k_x + k_y\gamma_2 k_y - 2i\{k_x\gamma_3 k_z\}\right]/(2m_0)$, $U = P_0 k_z/\sqrt{3}$, $V = P_0 k_-/\sqrt{6}$. In the above equation, $k = (k_\parallel, -\mathrm{i}\,\partial/\partial_\mathrm{z})$, $k_\pm = k_x \pm ik_y$, and $\{k_\alpha\gamma k_\beta\} = \left(k_\alpha\gamma k_\beta + k_\beta\gamma k_\alpha\right)/2$ $(\alpha, \beta = x, y, z)$. Here, $\mathbf{k}$=(k_x,k_y,k_z) is the momentum vector, $k_\parallel = \sqrt{k_\mathrm{x}^2 + k_\mathrm{y}^2}$ is the in-plane momentum vector, $\hbar$ is the reduced Planck constant, m_0 is the mass of a stationary electron and P_0 is the Kane irreducible matrix element of the momentum. The band parameters (depicted in Table 1) are assumed to be a piecewise function along the growth direction, $\gamma(z) = \sum_\mathrm{i}\gamma^i\left[\Theta\,(z - z_\mathrm{i}) - (z - z_{\mathrm{i}+1})\right]$, where $\Theta(z)$ is the Heaviside step function, z_i is the *i*-th interface of this system, and parameters γ^i (including E_g, E_c, E_v, Δ, A_c, γ_1, γ_2, and γ_3) are the bulk band parameters of the *i*-th layer, which can be derived from the Luttinger parameters and the effective mass[1]. We consider an in-plane magnetic field along the *y* direction *B* = (0, $B_\parallel$, 0). In the presence of an external in-plane magnetic field, the canonical momentum is **P** = **p** + *e***A**, where **A** = ($B_\parallel$ *z*, 0, 0) is the vector potential in the Landau gauge.

For an InAs/GaSb double layer quantum well structure, the lowest conduction subband in the InAs layer overlaps with the highest valence subband in the GaSb layer. Electrons can transfer from GaSb to InAs, inducing an internal electrostatic potential $V_{\text{in}}(z)$. Therefore, the total Hamiltonian becomes $H(\mathbf{k}) = H_0(\mathbf{k}) + V_{\text{in}}(z)$. The electronic structure of the quantum well system is obtained by solving the Schrödinger equation $H(\mathbf{k})|\Psi_s(\mathbf{k})\rangle = E(\mathbf{k})|\Psi_s(\mathbf{k})\rangle$, where s is the index of the subband and $|\Psi_s(\mathbf{k})\rangle = e^{ik_{\parallel}\cdot\rho}[\varphi_1^s(z), \varphi_2^s(z), \cdots, \varphi_8^s(z),]^T$ is the envelope function. We solved the Schrödinger equation by expanding the envelope function $\varphi_n^s(z)$ in a series of plane waves,

$$\varphi_n^s(z) = \frac{1}{\sqrt{L}} \sum_{m=-N}^{N} c_{nm}^s e^{ik_m z} \quad (4)$$

where k_m=$2m\pi/L$ and L is the total length of the structure. The internal electrostatic potential is determined by the Poisson equation $\nabla_z \varepsilon(z) \nabla_z V_{in}(z) = -(\rho_e(\text{z}) + \rho_h(\text{z}))$, where $\rho_e(\text{z})$ and $\rho_h(z)$ are the electron and hole charge densities, respectively. $\varepsilon(z)$ is the static dielectric constant. And $\rho_e(\text{z})$ and $\rho_h(\text{z})$ can be derived from the envelope function,

$$\rho_e(z) = -\frac{e}{(2\pi)^2} \sum_s \int \sum_{n=1,2} |\varphi_n^s|^2 f_F(E_s) dk_{\parallel}$$

$$\rho_h(z) = \frac{e}{(2\pi)^2} \sum_s \int \sum_{n=3,4,\cdots,8} |\varphi_n^s|^2 [1 - f_F(E_s)] dk_{\parallel} \quad (5)$$

where $f_F(E_s)$ is the Fermi distribution function and e is the absolute value of the electron charge. The summations $\Sigma_{\text{n=1,2}}$ and $\Sigma_{\text{n=3,4,…,8}}$ run over the electron components φ_1^s, φ_2^s and hole components $\varphi_1^s, \varphi_2^s, \cdots, \varphi_8^s$, respectively. The Fermi level is determined by the charge

neutrality condition $\int_0^L [\rho_e(z) + \rho_e(z)] dz = 0$. We obtained the eigenstates and eigen energies of the quantum well system numerically by solving the Schrödinger and Poisson equations self-consistently.

Supplementary Note 7: In-plane magnetic fields decoupling the conduction and valance bands in momentum space

We consider an in-plane magnetic field along the y direction, $\mathbf{B} = (0, B_{\parallel}, 0)$. In the presence of an external in-plane magnetic field, the canonical momentum is $\mathbf{P} = \mathbf{p} + e\mathbf{A}$, where $\mathbf{A} = (B_{\parallel}\, z, 0, 0)$ is the vector potential in the Landau gauge.

In a type-II InAs/GaSb quantum well structure, electrons are confined in the InAs layer while holes are localized in the GaSb layer. Since electron and hole bands are spatially separated into two layers by around a few nanometers depending on the thicknesses of the InAs and GaSb layers, the orbital effect of an in-plane magnetic field can induce an opposite shift between the electron and hole bands in momentum space (along k_x direction in our case). This can be understood from the formula of the canonical momentum $\mathbf{P} = \mathbf{p} + e\mathbf{A}$ in the Hamiltonian, by noticing that the charge e has opposite signs or electron and hole, which leads to the separation of electron and hole bands in momentum space. In Fig. 4f, we plot the energy dispersion of the conduction and valence bands under different in-plane magnetic fields $B_{\parallel}$. It is found that the two parabolas can be completely separated in momentum space, undergoing a semiconductor-to-semimetal transition near $B_{\parallel} = 18$ T. When the in-plane magnetic field $B_{\parallel}$ exceeds 18 T, the hybridization between the conduction and valance bands can be largely suppressed near the two parabolas. Such suppression was observed experimentally before. Supplementary Figure 10 describes the contour plot of subband components as a function of magnetic field and k_x, which can further prove the suppression of hybridization between electron and hole states under strong magnetic fields $B_{\parallel}$ near the

two parabolas. For example, when the magnetic field is very strong, e.g., $B_{\parallel}$ =35 T, the first and second conduction subbands (CB1 and CB2) are pure electron states and the first and second valance subbands (VB1 and VB2) are pure heavy hole states.

Supplementary Note 8: 35 T gap in a small perpendicular magnetic field

It was necessary to study the gap for low n_0 in a small perpendicular field while $B_{\parallel}$ was nearly 35 T. In a 35 T magnetic field, by rotating the device by small degrees, ±0.5 T, ±1 T, and ±2 T perpendicular fields were applied to a Corbino device from wafer A, with $B_{\parallel}$ still close to 35 T. As shown in Supplementary Figure 11, the conductance of electrons and holes were sensitive to the perpendicular field while the EI gap stayed the same and were independent of the perpendicular field. This confirms that the observed nontrivial EI gap under 35 T is not caused by any perpendicular field from small and accidental derivation from the plane.

Supplementary Note 9: In-plane magnetic field alignment

Through the in-situ Hall signal, the device surface could be aligned to parallel to the magnetic field with a deviation angle less than 0.04 degrees.

Supplementary references:

1. Vurgaftman, I., Meyer, J. R. & Ram-Mohan, L. R. Band parameters for III-V compound semiconductors and their alloys. *J. Appl. Phys.* **89**, 5815–5875 (2001).
2. Du, L. J., Knez, I., Sullivan, G. & Du, R. R. Robust helical edge transport in gated InAs/GaSb bilayers. *Phys. Rev. Lett.* **114**, 096802 (2015).
3. Suzuki, K., Harada, Y., Onomitsu, K. & Muraki, K. Gate-controlled semimetal-topological insulator transition in an InAs/GaSb heterostructure, *Phys. Rev. B* **91**, 245309 (2015).
4. Knez, I., Du, R. R. & Sullivan, G. Finite conductivity in mesoscopic Hall bars of inverted InAs/GaSb quantum wells, *Phys. Rev. B* **81**, 201301 (2010)
5. Zittartz, J. Anisotropy Effects in the Excitonic Insulator, *Phys. Rev.* **162**, 752-758 (1967).
6. Buttiker, M. Absence of backscattering in the quantum Hall effect in multiprobe conductors, *Phys. Rev. B* **38**, 9375-9389 (1988).
7. Li, J., Yang, W. & Chang, K. Spin states in InAs/AlSb/GaSb semiconductor quantum wells, *Phys. Rev. B* **80**, 035303 (2009).
